# Supplementary material for: ‘I Was Smoking a Lot More during Lockdown Because I Can’: A Qualitative Study of How UK Smokers Responded to the Covid-19 Lockdown
Source: Int J Environ Res Public Health. 2021 May 28;18(11):5816. doi: 10.3390/ijerph18115816 (PMC8198893; doi:10.3390/ijerph18115816)
Supplement: Supplementary file 1 [file ijerph-18-05816-s001.zip › ijerph-1208929-supplementary.pdf]

Table S1. Study sample plan.

Sample Variable		Target Proportions by Sub-Group	Full Sample [1] (n = 32)	Smoking Household Sub-Sample (n = 25)
Gender	Female	50%	16 (50%)	12 (48%)
	Male	50%	16 (50%)	13 (52%)
Age	Working age (21-64)	75%	25 (78%)	19 (76%)
	Retirement age (65+)	25%	7 (22%)	6 (24%)
Smoking status[2]	Smoking household	80%	25 (78%)	25 (100%)
	Non-smoking household	20%	7 (22%)	NA (0%)
Access to private outdoor space[3]	Access to private outdoor space	50%	19 (59%)	14 (56%)
	No access to private outdoor space	50%	13 (41%)	11 (44%)
Household size	Single person household	20%	6 (19%)	4 (16%)
	Multi-person household	80%	24 (81%)	21 (84%)
Children[4]	Children under 18	50%	16 (50%)	11 (44%)
	No children under 18	50%	16 (50%)	14 (56%)
Effect of lockdown on work-life status[5]	Changed	75% (66%)	23 (72%)	15 (60%)
	Unchanged	25% (33%)	9 (28%)	10 (40%)

(1) Quota sampling strategy was applied when selecting the full interview sample, which included both smoking and non-smoking households; (2) Smoking status when assessed at household level. Smoking households defined as containing one or more smokers, and non-smoking households as not containing anyone who smokes; (3) Private outdoor spaces included gardens, yards and private balconies; (4) Households with children were defined as those with at least one child aged under 18 living at home; (5) Work-life status was categorised as 'changed' due to lockdown, (e.g. furloughed, forced to work from home, made unemployed, forced to shield at home) and 'unchanged' during lockdown (e.g. carried on working in usual out of home setting, carried on in carer role, remained unemployed, remained non-working). [1] Social grade was based on household chief income earner: A—higher managerial, professional; B—intermediate managerial, professional; C1—supervisory or clerical, junior managerial, student; C2—skilled manual worker; D—semi or unskilled manual work, casual worker; E—retired, unemployed, not-working due to long-term sickness, full-time carer, homemaker.
